# Update on ICH E14/S7B Cardiac Safety Regulations: The Expanded Role of Preclinical Assays and the “Double‐Negative” Scenario

**DOI:** 10.1002/cpdd.1003

**Published:** 2021-07-31

**Authors:** Robert M. Lester

**Affiliations:** ^1^ Global Clinical Research Celerion Tempe Arizona USA

**Keywords:** cardiac liability, concentration response analysis, hERG, proarrhythmia risk, thorough QT study (TQT)

## Abstract

For nearly 2 decades, regulators have adopted a harmonized approach to drug development, which has succeeded in bringing new pharmaceuticals to market without significant cardiac liability. Ushered in by technological advancements and better understanding of cellular electrophysiology, the initial paradigm detailed in the 2005 International Conference for Harmonization E14 and S7B documents has undergone evolutionary changes designed to streamline drug development and improve regulatory decision‐making and product labeling. The intent of this review is to summarize the new US Food and Drug Administration (FDA) Question and Answer update from August 2020 and key messaging from a subsequent FDA webinar describing best practices for preclinical and clinical data integration into a QT risk prediction model.

In August 2020 the International Conference for Harmonization (ICH) Implementation Working Group (IWG) developed a draft guideline document titled “Clinical and Nonclinical Evaluation of QT/QTc Interval Prolongation and Proarrhythmic Potential: Questions and Answers.” This was followed by a US Food and Drug Administration (FDA) webinar in October 2020 addressing and clarifying some of the key issues enumerated in the IWG guidance and discussing new approaches for an integrated nonclinical‐clinical QT proarrhythmic risk assessment. The ICH has followed a 5‐step approach regarding updating the original ICH E14/S7B guidance to ensure safe and cost‐effective drugs are developed. These steps are building consensus among experts, adopting a draft guideline, soliciting public input and instituting revisions to the document, formulating a new harmonized guideline, and implementing the guideline, which is currently targeted for release around 2022. The webinar represented the third step in this process, with public feedback having been requested through November 2020.

A number of excellent reviews concerning proarrhythmic risk have been published whose focus was to describe the individual preclinical assays, clinical studies, and analysis schemes involved in assessing a compound's effect on ventricular repolarization.^1^, ^2^, ^3^, ^4^ As the science and technology in the field of cardiac safety have evolved, so has the regulatory landscape. As such, the purpose of this review is to highlight the principal regulatory changes from the IWG publication and FDA webinar regarding the expanded role of concentration response analysis (CRA) and preclinical assays designed to evaluate the proarrhythmic potential of compounds when a substitute for a thorough QT study (TQT) study is sought or when a dedicated TQT study is not feasible. As a by‐product of these changes, there is a defined strategy to integrate preclinical and clinical study results into a risk prediction model, thereby improving QT liability determination, reducing the number of stand‐alone TQT studies, and informing regulatory decision‐making and drug labeling.

## Historical Background: The QT and hERG‐Centric Models

The QT interval on the electrocardiogram (ECG) has garnered the attention of clinicians for more than 60 years beginning in 1957 with descriptions of sudden death in children who had associated deafness (Jervell and Lange‐Nielsen syndrome).^5^ This was followed in the ensuing years by multiple reports of lethal ventricular arrhythmias in patients who presented with QT prolongation secondary to antiarrhythmic medications including the first description in 1966 by Desertenne^6^ of a unique polymorphic form of ventricular tachycardia termed torsades de pointes (TdP). However, it was not until the 1990s that regulators began to focus on QT prolongation following the death of otherwise healthy individuals treated with the antihistamine Seldane. Thereafter, in response to fatal cases of ventricular arrhythmias and TdP primarily related to nonantiarrhythmic drugs, in May 2005 the ICH drafted a document E14 titled “A New Regulatory Guidance on the Clinical Evaluation of QT/QTc Interval Prolongation and Proarrhythmic Potential.” The primary role of this document was to help guide the need for ECG surveillance in phase 3 trials and not to definitively determine TdP risk.

The in vitro electrophysiologic substrate for TdP is typically block of the human ether‐à‐go‐go related gene (hERG) channel, which governs movement of the predominant outward delayed rectifier potassium current known as Ikr and results in altered ventricular repolarization. Inhibition of this channel results in an increase in action potential duration, which manifests in vivo on the ECG as QT prolongation. In addition, hERG block may increase the probability of l‐type calcium current producing early afterdepolarizations that can provide the substrate to trigger TdP. Since TdP is a very rare event in drug development, a surrogate end point, the QT interval, became the focus of the E14 guidance and formed the basis for the recommendation that a dedicated TQT study be performed on all new pharmaceuticals that have systemic exposure to assess QT liability. Monoclonal antibodies and large proteins that have low likelihood of affecting cardiac ion channels are typically exempted from this requirement, as would many dermatologic preparations that do not generate systemic exposures and do not cross cardiac cell membranes. Oncologic agents for patients with advanced cancer, per ICH S9, would similarly not be required to undergo a stand‐alone cardiovascular safety study

That same month in 2005, the ICH also published S7B, which refers to “The Nonclinical Evaluation of the Potential for Delayed Ventricular Repolarization (QT interval prolongation) by Human Pharmaceuticals.” The hallmarks of this publication included evaluating potential block of the hERG channel coupled with in vivo nonrodent mammalian animal studies to assess their effects on ventricular repolarization. In addition, action potential duration in Purkinje fibers or ventricular wedge preparations was also suggested. Unfortunately, QT prolongation and hERG block are both imperfect predictive markers for TdP risk, and these preclinical assays have routinely been marginalized and interpreted independent of clinical trial findings. They have been primarily used to sanction the safety of proceeding with the test article in human subjects, whereas clinical studies were the principal drivers of QT liability and proarrhythmic assessment.

The seminal ICH E14 document has been the subject of multiple question‐and‐answer (Q&A) commentaries published in 2008, 2012 (Q&A R1), 2014 (Q&A R2), and 2015 (Q&A R3). The latter document reviewed the role of CRA in regulatory decision‐making (question 5.1) and alternative study designs when a conventional TQT study could not be undertaken (question 6.1). Most recently, the IWG Q&A document from August 2020 revised initial responses to questions 5.1 and 6.1 from 2015 to provide more clarity and address ambiguities while adding a new section on questions and answers pertaining to S7B preclinical assays.

**Figure 1:**
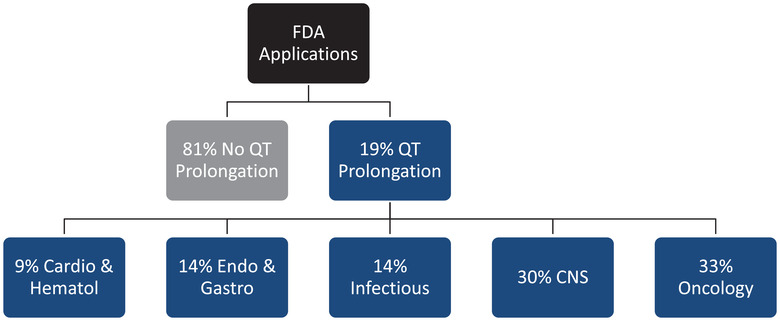
Observed QT response and therapeutic area during FDA application submission. Data presented during FDA Webcast “New Approaches for an Integrated Nonclinical‐Clinical QT/Proarrhythmic Risk Assessment,” October 15‐16, 2020.

Approximately 19% of recent drug studies reviewed by the FDA have demonstrated QT prolongation, as noted in Figure 1, and spanned a range of different therapeutic areas. The original ICH draft documents have been highly successful in preventing drugs with unknown torsadagenic risk from coming to market. However, a number of potentially beneficial compounds may have been prematurely terminated in their development because of hERG block or an in vivo QT safety signal despite the absence of TdP or any other serious ventricular arrhythmias associated with the test article. This underscores that QT studies, although highly sensitive, are not very specific, and their positive predictive value for proarrhythmia risk is modest. The same holds true for the hERG assay in large measure because of its promiscuity for agents that block the potassium channel but do not contribute to arrhythmia occurrence.

In an effort to lessen premature drug discontinuation and reduce the high number of costly and resource‐intensive TQT studies that have been conducted in recent years, several initiatives have been introduced facilitated by our understanding of cellular electrophysiology in conjunction with advances in laboratory technology. In 2013, the Comprehensive In Vitro Proarrhythmic Assay (CiPA) was promulgated by a public‐private collaboration that described a suite of 4 predominantly nonclinical assays as a way to mechanistically profile a compound's proarrhythmic risk and shift focus away from the QT interval.^7^ These assays laid the foundational elements present in the recent S7B update.

1. Obtain information on multiple ion channels beyond hERG/Ikr.
2. Develop in silico proarrhythmic risk models based on the data gleaned from the ion channel assays employing human ventricular action potential reconstructions.
3. Utilize human‐induced pluripotent stem cell cardiac myocytes (hiPSC‐CMs) to assess arrhythmogenicity of the new pharmaceutical.
4. Measure the biomarker J‐T peak interval on clinically acquired ECGs to assess late inward Na^+^ and l‐type Ca^++^ channel currents, which may mitigate risk of TdP in the setting of a prolonged QT interval.

In 2014, a pilot study involving the FDA and Cardiac Safety Research Consortium demonstrated that it was feasible to use CRA to detect a slight QT effect in a small group of individuals who participated in a dose‐escalation protocol.^8^ They found that the QTc results mimicked the known QTc effects of the 6 tested drugs that had been documented in prior TQT studies.^8^ Based on this finding, the E14 2015 Q&A R3 document profiled the emerging role of CRA as a primary analysis tool as a way to obtain a “waiver or substitute” for a conventional TQT study. However, the 2015 guidance lacked sufficient prescriptive detail, and consequently the role of CRA was further refined and the requisite components of this approach were described in a 2018 white paper by Garnett et al.^9^

The CiPA paradigm was a beneficial addition to the S7B guidance and reflected a shift away from a traditional QT‐centric focus to a pathophysiologic paradigm of arrhythmogenesis, whereas CRA was viewed as an important alternative to the original E14 primary statistical analysis tool known as the Intersection Union Test (IUT). CiPA was also noteworthy in that it provided a more scientifically rigorous schema that underscored the need to more fully link and integrate preclinical data with human studies into a working proarrhythmic risk model. The culmination of this need was the IWG August 2020 question‐and‐answer publication followed by the FDA webinar in October and most recently, the report from the 35 members of the ICH E14/S7B Industry Support Group in 2021.^10^

## Updated Responses to Questions Regarding Study Designs of New Pharmaceuticals Based on the Original 2015 Q&A (R3) Draft Document

QUESTION #5.1: What is the role of concentration‐response analysis in QTc assessment, and what are the design pathways in which a positive control could be waived in early‐phase studies?

ANSWER #5.1: CONCENTRATION‐RESPONSE ANALYSIS. CRA has been deployed for many years as a secondary analysis tool in conventional TQT studies, where the by‐time point or IUT was the primary analysis modality to assess QT risk. However, CRA is now being used and increasingly accepted as an alternative to the IUT to evaluate the presence of a QTc safety signal and can be part of the body of evidence regarding arrhythmia risk prediction and decision‐making. This enhanced role has been profiled in reviews by Grenier et al^11^ and Garnett and colleagues.^9^

When CRA is used to estimate a drug's risk, the upper bound of the 2‐sided 90% confidence interval around the estimated maximal effect on ΔQTc should be less than 10 milliseconds at the highest clinically relevant exposure to conclude that the investigative product is low risk and expanded ECG collection in later‐phase studies is not essential. The growing popularity of CRA as the primary analysis tool for QT risk assessment during the past 5 years is evidenced by a reduction in submitted TQT studies from 62% to 34% with a corresponding increase in first‐in‐human protocols from 10% to 42% (unpublished data presented during Pharmaceutical and Bioscience Society Webcast 2021).

CRA is most often undertaken in first‐in‐human single‐ and/or multiple‐dose escalation studies aiming for an exposure at either C_max_ and/or steady state that exceeds the maximal therapeutic dose that is essential for discerning a small QT effect. The acquisition of high‐quality ECGs is also necessary for this type of analysis, whereas pooling of data across studies is acceptable assuming there is no heterogeneity that would introduce bias in the interpretation of results. Evaluating the time course and magnitude of QT prolongation as well as categorical outliers are additional elements that need to be incorporated into proarrhythmic risk assessment. Furthermore, the model and methods used for analysis need to be specified along with tests for hysteresis and goodness of fit.

A potential shortcoming of CRA is that on occasion the model may not fully explain the observed QT effects. Changes in ventricular repolarization may be independent of drug exposure secondary to protein trafficking and ion channel gating, abnormal protein synthesis, nonionic mechanisms, or autonomic changes. In addition, delayed QT effects because of hysteresis of the parent compound or metabolites and alterations in glycemia homeostasis may also contribute to QT changes not identified with CRA.

**Figure 2:**
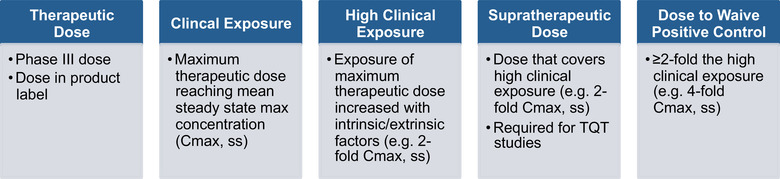
Defining relevant doses and clinical exposure. Adapted from FDA Webcast “New Approaches for an Integrated Nonclinical‐Clinical QT/Proarrhythmic Risk Assessment,” October 15‐16, 2020.

ANSWER #5.1: WAIVING A POSITIVE CONTROL. There are 2 main pathways presented for conducting studies without a positive control using CRA, and to understand these scenarios, it is incumbent to define the relevant dose and exposure terms (Figure 2).

1. A positive control could be avoided if the study design achieved an exposure of the test compound, which is a multiple of the high clinical exposure (≥2‐fold) accounting for both intrinsic (eg, renal or hepatic dysfunction or genetic polymorphisms) and extrinsic factors (eg, drug‐drug interactions). This circumstance has been previously described in the 2018 white paper by Garnett et al.^9^
2. The second pathway to avoid a positive control involves the performance of best practice‐designed preclinical in vitro hERG channel testing and in vivo nonrodent telemeterized animal studies. In the event that *both* of these assays are deemed negative for altered ventricular repolarization and QT prolongation, respectively, the so‐called “double‐negative”, a positive control would not be required if only the high clinical exposure scenario is fully covered.

This latter pathway represents a *new initiative* that supports lower clinical exposures than previously recommended and is a departure from typical FDA recommendations of performing a TQT study when sufficiently high exposures cannot be attained because of tolerability, safety, or saturation absorption concerns. The potential impact of this initiative in reducing the number of dedicated TQT studies is supported by data from Strauss and colleagues, who found that between 2016 and 2020, only 42% of submitted QT studies to the FDA covered twice the high clinical exposure scenario and would not have necessitated further QT study if the “double‐negative” criteria had been met.^12^

To fulfill the “double‐negative” requirement and to elicit confidence in these preclinical assays conferring “low likelihood” of risk, the following criteria have been proposed:

1. There is a robust hERG safety margin comparing the test compound with a library of compounds with defined TdP risk, all tested under the same experimental conditions.
2. The in vivo animal study being of “sufficient sensitivity conducted at exposures of parent compound and human‐specific major metabolites that exceed clinical exposures” does not reveal QTc prolongation of a magnitude that would be seen in traditional QT studies.

QUESTION #6.1: What adaptive study designs are acceptable to profile a compound's QT risk and proarrhythmic potential when a traditional TQT with a supratherapeutic dose, placebo, and positive control cannot be implemented or safety concerns preclude its administration to healthy individuals (eg, with oncologic agents)?

ANSWER #6.1: There are 3 basic criteria required to proclaim that a drug has a “low likelihood of proarrhythmic effects” when integrating clinical and nonclinical data for drugs that cannot undergo a dedicated TQT study:

1. The hERG patch clamp assay utilizing best practices demonstrates a safety margin of the test compound that exceeds the known TdP threshold of reference drugs employing the same assay for both data sets AND,
2. The animal in vivo QT study being sufficiently powered to identify QTc prolongation to the same degree as would be seen in a conventional TQT trial and employing best practices shows no QTc prolongation in which both the parent and any relevant metabolites cover the worst‐case clinical exposure scenario.
3. Last, to declare that the compound has low proarrhythmic risk, high‐quality ECGs should be obtained in patients in which that the upper bound of the 2‐sided 90% confidence interval around the estimated maximal mean effect on ΔQTc is less than 10 milliseconds without any significant imbalance in subjects exceeding outlier values. In addition, the prevalence of adverse events in the cardiac safety database for that drug does not indicate a propensity for arrhythmia events.

The first 2 criteria constitute a “double‐negative” nonclinical assessment and support alternative study designs for proarrhythmic risk assessment in lieu of a formal TQT study. This is a *new initiative* that may be applicable in a subset of QT studies submitted to the FDA and will likely have an important impact on the development strategy for both oncologic and other investigational products, as approximately 25% of QT studies submitted to the FDA since 2016 would fall in this category.^12^ Equally important is that the original 6.1 Q&A for clinical studies without a positive control focuses on excluding QTc effects defined as <10‐millisecond QTc prolongation using either the IUT or exposure‐response analysis. In contrast, the revised 6.1 Q&A centers on proarrhythmic risk. In this case, if the upper bound of the 2‐sided 90% confidence interval around the mean ΔQTc estimate is <10 milliseconds, then there is “low likelihood of proarrhythmic risk.” Finally, a major distinction between Q&A 5.1 and 6.1 is that the latter is more highly powered similar to a traditional TQT study and does not always rely on exposures that may exceed the anticipated therapeutic dose.

There are a number of caveats regarding the “double‐negative” assessment, and the guidance does not offer sufficient prescriptive recommendations for each assay to facilitate decision‐making. For example, what is considered an acceptable hERG safety margin, and what are the essential design and analysis elements to ensure that an in vivo study is adequately powered and is truly negative? As such, the criteria for excluding a false‐negative assay need to be delineated so that sponsors can have assurance that they have fulfilled the “double‐negative” scenario and can safely move forward with their compound's development program.

As a correlate, interpreting results in a binary manner as either positive or negative is simplistic, as indeterminate or low‐risk findings may be present. In these cases, it is unclear which, if any additional preclinical assays should be undertaken or whether optional ECG biomarker evaluation (eg, J‐T peak measurement) would be helpful. Moreover, low proarrhythmic risk does not translate into no risk, and it remains to be defined what constitutes a drug having “low likelihood proarrhythmic risk” and how this impacts the development strategy including acquisition of ECGs in later‐phase studies and product labeling. Finally, there is a paucity of data concerning when, how often, and which of the S7B assays should be undertaken and how each is weighted in the totality of evidence supporting drug submission. In this regard, it is of interest that recent commentary by Vargas et al^10^ proclaimed that the in vivo animal data are a “stronger” predictor of TdP liability than the in vitro hERG assay, whereas the relative contribution of other preclinical assays and the J‐T peak biomarker to arrhythmia risk are not specifically delineated.

A final issue is that there is mention of “confounding” heart rate effects as part of the integrated risk evaluation and a threshold value of >20 beats per minute (bpm) was quoted, which may require subject‐specific correction factors or other methods to inform risk. However, a recent publication cites a threshold of ±10 bpm as significant where fixed heart rate QTc corrections can be “problematic” and individual heart rate corrections should be considered along with assessment of QT/RR hysteresis.^13^ This apparent discrepancy in thresholds, especially when applied to smaller trials, should be clarified for both in vivo and first‐in‐human studies. Finally, the algorithm used by clinical research organizations for calculation of a subject‐specific individual QT correction factor is highly variable and consensus within the industry remains an elusive goal.

## ICH S7B Questions and Answers

**Figure 3:**
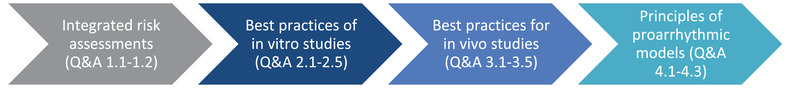
Four key topics covered in S7B. Q&A section headings as listed in E14 and S7B clinical and nonclinical evaluation of QT/QTc interval prolongation and proarrhythmic potential—Questions and Answers. Draft. 2020.

There were 4 major topics covered in the S7B document pertaining to the role of preclinical assays in assessing ventricular repolarization, requisite elements to standardize the in vitro and in vivo tests, proper formatting of results, and how best to integrate this information with clinical data into a proarrhythmic risk prediction model (Figure 3).

### Integrated Risk Assessment

In recent years our knowledge about cellular electrophysiology and the substrate for ventricular arrhythmias and TdP has increased considerably. For example, drugs such as verapamil and amiodarone that inhibit the hERG channel are not viewed as proarrhythmic, whereas other compounds such as pentamidine and arsenic trioxide that do not directly block hERG but increase the QTc interval can lead to dangerous arrhythmias.^2^ Abnormalities in protein trafficking and synthesis, reduction in the number of mature channels, effects on other repolarizing ionic currents such as IKs, increases in late Na^+^ current, metabolites that interfere with ion channels, and nonionic mechanisms have all been identified and linked to TdP. As such, the need for best practice in vivo animal data is critical to complement the patch clamp studies and ascertain whether a “double‐negative” is present, which would inform the development scheme going forward. In cases in which the “double‐negative” is not present in core assays because of either hERG block or QT prolongation, then the totality of evidence from optional supplemental preclinical assays needs to be integrated with human studies to profile a compound's proarrhythmic potential.

As stated in the Q&A, the best‐practice considerations are designed to be used by sponsors when an integrated risk assessment with clinical data is desired as outlined in Q&A 5.1 and 6.1. Otherwise, if the preclinical data are aimed at informing first‐in‐human drug design, screening for signals that would influence go‐no‐go decisions or aiding in commercialization, then the best‐practice considerations need not be undertaken.

### Principles of Proarrhythmia Risk Models

Although model input may vary between models, the output should be similar between them and therefore be beneficial in drafting a compound's predisposition to QT prolongation and TdP. Moreover, the magnitude of risk should be independent of which model is employed, acknowledging that human arrhythmia risk may not be precisely predicted from preclinical experimental data. The following are the general principles required for all proarrhythmic risk prediction models and should be integrated with clinical information.

1. Defined end point(s);
2. Defined scope and limitations of the model including the experimental protocols utilized;
3. Prespecified analysis plan;
4. Detailed algorithm of how experimental data will be incorporated into proarrhythmic risk;
5. Variability in model input should be quantified, which might influence predicted risk;
6. Mechanistic interpretation of the model about how this would translate into arrhythmia prediction.

### In Vitro Studies: Best‐Practice Considerations

When referring to in vitro studies, the IKr/hERG patch clamp assay is the gold standard to determine if hERG channel block poses a risk of delaying or altering ventricular repolarization. There have been a variety of protocols and practices leading to significant data variability, and former guidance lacked enough critical detail to permit consistent and uniform performance of the assay. Based on recent improvements in modeling and better understanding of the molecular mechanisms involved in hERG blockade, there is now the opportunity to develop a systematic approach in executing patch clamp studies to assess the parent compound and any relevant metabolite's effects on IKr. To this end, careful attention to detail is essential when performing the hERG study, as there are a number of experimental conditions that can affect the results of the assay (Table 1).

**Table 1:** Key Experimental Conditions and Considerations for In Vitro Assays

Condition	Comment
hERG assay	
*Recording temperature*	Goal is to execute at physiologic temperatures (35°C‐37°C)
*Voltage protocol*	Should approximate ventricular action potential ionic currents minus background residual current
*Recording quality*	Seal resistance to ensure stability of ionic currents
*Primary end‐point measures*	Including the IC_50_ and Hill coefficient data
*Data summary*	Cell‐specific inhibition information at different exposures including free C_max_ at steady state and highest total exposure
*Concentration verification*	Document the exposures to which each cell was exposed
*Positive and negative controls*	Assay sensitivity needs confirmation at 2 or more concentrations covering 20%‐80% block
hiPSC‐CM assay	
*Biological preparation*	Source of cells and baseline electrophysiologic characteristics should be enumerated
*Technology platform*	Methodology utilized to assess transmembrane potentials including recording temperature, beating rate of the preparation
*Important considerations*	High‐fidelity recordings, pacing protocol when applicable, characterize drug exposure/concentration
*Assay sensitivity*	Calibration of the preparation with use of concentration‐response curves with known agents that can inhibit IKr and also evaluating late‐depolarizing inward l‐type calcium and sodium currents that shorten repolarization

Adapted from E14 and S7B Clinical and Nonclinical Evaluation of QT/QTc Interval Prolongation and Proarrhythmic Potential—Questions and Answers. Draft. 2020.

hERG block potency is the amount of free drug in the steady state, which inhibits the potassium current by 50% (IC_50_), and this can be compared with the estimated clinically relevant exposure to calculate a safety margin. This safety margin can then be benchmarked against drugs with known TdP risk to provide a human safety margin estimate. A robust margin of >30 was previously proposed by Redfern^14^ as optimal, although a recent analysis by Ridder and colleagues^15^ suggested a higher threshold margin may improve the hERG assay's predictive power. In the Ridder report, they evaluated the safety margin's specificity and sensitivity for predicting TdP by reviewing ambient (rather than physiologic) temperature patch clamp assay results from 13 prospective studies and compared these against the 28 drugs cited in the CiPA paradigm. They found that progressive increases in the margin beyond 50 increased sensitivity of the assay while decreasing its specificity. Although the current S7B document does not provide a specific safety margin threshold, it would appear that a margin between 30 and 50 would offer an acceptable safety buffer. Finally, the margin that would be considered safe also depends on factors including the solubility of the drug candidate, the underlying disease in the target population, and what risk tolerance would be acceptable if the drug was administered to affected individuals.

hiPSC‐CM or acutely isolated human ventricular myocytes are not required for all submissions but can be used to determine the test article's effect on multiple ionic currents. Both transmembrane action potential and extracellular field potential duration can be measured to identify prodromal markers of ventricular arrhythmias such as early afterdepolarizations or triggered activity. These studies can add to the entirety of evidence in assessing the proclivity of a compound to induce arrhythmias and clarify the significance of positive signals that may have been obtained from other assays. The most important considerations related to myocyte studies are listed in Table 1.

### In Vivo Studies: Best‐Practice Considerations

In vivo studies have been part of core battery assays for the past 16 years. During this period lessons have been learned regarding study design, performance of hiPSC‐CM assays and reporting of assay results. Nonetheless, there remains considerable variability regarding which nonrodent species is studied, whether the study is sufficiently powered to determine a QT effect, whether adequate exposures are achieved, what is the appropriate heart rate‐QT correction factor to use, and how best to present and incorporate the findings into clinical decision‐making. To address these issues and promote standardization, a number of important elements were outlined in the latest guidance (see Table 2).

**Table 2:** Summary of In Vivo Study Considerations

Consideration	Comment
*Nonrodent animals*	Telemeterized nonrodent animal species that are freely moving and the same species as used for toxicity studies
*Drug exposure*	Drug exposures should ideally include or exceed therapeutic concentrations and cover the highest clinical exposure if used to support Q&A 5.1 and 6.1
*Modeling*	Exposure‐response modeling to assess QTc effects should characterize both parent and any metabolites
*Heart rate correction*	Determination of heart rate correction factors to show independence of QTc from RR intervals
*Assay sensitivity*	Need to demonstrate and validate assay sensitivity with positive control or historical positive data that are adequately powered
*Data submission*	Submission of supporting tables, figures, and listings of all relevant pharmacokinetic and pharmacodynamics data

Adapted from E14 and S7B Clinical and Nonclinical Evaluation of QT/QTc Interval Prolongation and Proarrhythmic Potential—Questions and Answers. Draft. 2020.

## Summary and Future Considerations

E14 was intended to help inform the need for ECG monitoring in later‐phase trials, although its QT‐centric focus lacked mechanistic insight into the pathogenesis of ventricular arrhythmias and TdP. Conversely, S7B was devised to inform a safety signal that might influence the development strategy of the sponsor while complementing clinical QT studies. However, S7B fell short in a variety of prescriptive details regarding in vitro and in vivo assays.

The most recent IWG Q&A document is a welcome addition in the evolution of drug development and safety. It is noteworthy in introducing the concept of a “double‐negative” preclinical assessment with core hERG and in vivo animal studies to support how trials can be structured without a positive control (Q&A 5.1) and what alternative study designs in special cases (Q&A 6.1) would be acceptable when a traditional TQT study cannot safely be performed. Most importantly, the Q&A paper serves to link often marginalized and independently analyzed preclinical information with clinical data into an integrated proarrhythmic risk prediction model. Moreover, it provides important specifics regarding the conduct and reporting of preclinical assays so as to harmonize methodology and reduce variability. Last, it broadens the circumstances and provides insights about how to structure and analyze studies using CRA that would suffice as a substitute for a conventional TQT study.

Despite these advances, there are still a number of unanswered questions and gaps in information that will undoubtedly be addressed as regulators refine the ICH documents and construct a comprehensive database of results and metrics from completed protocols to better inform all stakeholders. Chief among these is:

1. How does a sponsor ascertain that the preclinical package is adequate to fulfill regulatory requirements, and, conversely, how do they determine if it is not acceptable?
2. There needs to be continued pivoting away from a QT‐centric focus given its lack of positive predictive value for TdP and malignant ventricular arrhythmias in concert with avoiding the binary interpretation of assay information and adoption of a graded continuum of risk based on the totality of preclinical and clinical data.
3. The guidance gives significant weight to hERG and in vivo studies as part of the “double‐negative” assessment, although other nonrodent in vitro and in vivo models and clinical assays may be helpful to characterize proarrhythmic risk assuming they are executed using standardized best practices. For example, these additional assays might include ventricular wedge preparations, methoxamine‐sensitized rabbit models for TdP,^16^ exploring non hERG mechanisms of QTc prolongation, and ECG biomarker analysis of transmural dispersion of ventricular repolarization (Tpeak‐Tend) and T‐wave morphology patterns.
4. It would be of interest for regulators to provide metrics concerning the number of cases in which the “double‐negative” scenario has been accepted or rejected. In cases in which the “double‐negative” is not fulfilled or preclinical assay results are discordant, subsequent recommendations by the agency would be enlightening; acknowledging this would presumably be done on a case‐by‐case basis.

As there is a desire to reduce the number of dedicated TQT studies, how often and under what circumstances is a stand‐alone study still advisable and recommended by regulators? The guidance leaves open the question of how to identify and define drugs with “low likelihood of proarrhythmic risk” and what additional evaluation, if any, needs to be undertaken prior to approval to assess the drug's arrhythmia liability. In addition, how do sponsors determine which clinical research organizations are optimally positioned to perform preclinical assays and what steps are necessary to ensure that best practices are followed and at what cost? What is the optimal approach to assess chimeric moieties for their proarrhythmic potential?

Finally, the recent review by Vargas et al^10^ questioned the value of carrying out enhanced clinical studies for small chemical molecules when best‐practice preclinical assays either demonstrate a small or no cardiac safety signal. They systematically reviewed published data from multiple sources where QTc prolongation and TdP liability information was available and determined the probability for predicting QTc prolongation and TdP from different core assay scenarios. They found that when both assays were negative, the predictive risk for QTc prolongation was 3.8% and for TdP liability was approximately 0.1%. In view of the fact that the preponderance of reported QT clinical trials are deemed negative for delayed ventricular repolarization and consistent with the FDA's position that certain chemical entities be exempted from QT safety studies, the authors posited that conducting a healthy volunteer study when there is a “double‐negative” core assay scenario would likely not provide additional insights about risk. As such, only routine ECG surveillance in later‐stage trials might be sufficient in these cases for QT liability determination. However, it remains to be seen whether regulators will entertain accepting robust and comprehensive preclinical data for select investigational compounds and forego the requirement of performing a first‐in‐human or TQT clinical study.

## Conclusion

The most recent guidance is an important step in the evolutionary process of drug development by providing a more robust harmonized and integrated road map for all stakeholders to profile a compound's proarrhythmic potential while informing regulatory decision‐making and restrictive labeling. It remains to be determined whether the impact of this guidance will succeed in continued reduction in the number of stand‐alone TQT studies while conserving resources, accelerating development of novel drug candidates, and building on the ICH E14/S7B mission of preventing unsafe drugs from coming to market. Lastly, the ongoing efforts of the IWG to develop additional guidance regarding preclinical‐clinical data integration intended to inform ECG collection in later‐phase trials to clarify appropriate labeling of compounds with a positive QT signal and to address whether certain small molecules with limited bioavailability can circumvent undergoing a QT clinical study, are most appreciated.

## Conflicts of Interest

The author is a Celerion employee.

## Pertinent Regulatory Documents

Darpo B. Cardiac Safety from Preclinical to the Clinic: Industry and Regulatory Trends in T‐QT and Concentration‐QT Studies for Assessing QT Prolongation Risks (Webcast), March 23–24, 2021. Pharmaceutical and Bioscience Society. https://www.pbss.org/aspx/pevent2.aspx.

E14 Implementation Working Group. ICH E14 Guideline: The Clinical Evaluation of QT/QTc Interval Prolongation and Proarrhythmic Potential for Non‐Antiarrhythmic Drugs Questions & Answers (R3). 2015; International Conference on Harmonization of Technical Requirements for Registration of Pharmaceuticals for Human Use. https://database.ich.org/sites/default/files/E14_Q%26As_R3_Q%26As.pdf. Accessed February 13, 2021.

E14 Clinical Evaluation of QT/QTc Interval Prolongation for Non‐Antiarrhythmic Drugs – Questions and Answers (R3) Guidance for Industry. 2017; International Conference on Harmonization of Technical Requirements for Registration of Pharmaceuticals for Human Use. https://www.fda.gov/ucm/groups/fdagov‐public/@fdagov‐drugs‐gen/documents/document/ucm073161.pdf. Accessed February 13, 2021.

E14 and S7B Clinical and Nonclinical Evaluation of QT/QTc Interval Prolongation and Proarrhythmic Potential—Questions and Answers. Draft. 2020; International Conference on Harmonization of Technical Requirements for Registration of Pharmaceuticals for Human Use. https://www.fda.gov/media/142501/download. Accessed February 13, 2021.

Guidance for Industry S7B Nonclinical Evaluation of the Potential for Delayed Ventricular Repolarization (QT Interval Prolongation) by Human Pharmaceuticals. 2005; Food and Drug Administration. https://www.fda.gov/downloads/Drugs/GuidanceComplianceRegulatoryInformation/Guidances/ucm074963.pdf. Accessed February 13, 2021.

ICH Harmonised Tripartite Guideline: The Clinical Evaluation of QT/QTc Interval Prolongation and Proarrhythmic Potential for Non‐Antiarrhythmic Drugs E14. Current Step 4 Version. 2005; International Conference on Harmonization of Technical Requirements for Registration of Pharmaceuticals for Human Use. https://database.ich.org/sites/default/files/E14_Guideline.pdf. Accessed February 13, 2021.

New Approaches for an Integrated Nonclinical‐Clinical QT/Proarrhythmic Risk Assessment (Webcast). October 15–16, 2020. Food and Drug Administration. https://www.fda.gov/drugs/news-events-human-drugs/new-approaches-integrated-nonclinical-clinical-qtproarrhythmic-risk-assessment-10152020-10162020.

S9 Nonclinical Evaluation for Anticancer Pharmaceuticals. Guidance for Industry: 2010. International Conference on Harmonization of Technical Requirements for Registration of Pharmaceuticals for Human Use. https://www.fda.gov/media/73161/download. Accessed June 12, 2021.
